# Automated meta-analysis of the event-related potential (ERP) literature

**DOI:** 10.1038/s41598-022-05939-9

**Published:** 2022-02-03

**Authors:** Thomas Donoghue, Bradley Voytek

**Affiliations:** 1 Department of Cognitive Science, University of California, San Diego, La Jolla, USA; 2 Neurosciences Graduate Program, University of California, San Diego, La Jolla, USA; 3 Halıcıoğlu Data Science Institute, University of California, San Diego, La Jolla, USA

**Keywords:** Cognitive neuroscience, Human behaviour

## Abstract

Event-related potentials (ERPs) are a common approach for investigating the neural basis of cognition and disease. There exists a vast and growing literature of ERP-related articles, the scale of which motivates the need for efficient and systematic meta-analytic approaches for characterizing this research. Here we present an automated text-mining approach as a form of meta-analysis to examine the relationships between ERP terms, cognitive domains and clinical disorders. We curated dictionaries of terms, collected articles of interest, and measured co-occurrence probabilities in published articles between ERP components and cognitive and disorder terms. Collectively, this literature dataset allows for creating data-driven profiles for each ERP, examining key associations of each component, and comparing the similarity across components, ultimately allowing for characterizing patterns and associations between topics and components. Additionally, by examining large literature collections, novel analyses can be done, such as examining how ERPs of different latencies relate to different cognitive associations. This openly available dataset and project can be used both as a pedagogical tool, and as a method of inquiry into the previously hidden structure of the existing literature. This project also motivates the need for consistency in naming, and for developing a clear ontology of electrophysiological components.

## Introduction

Electroencephalography (EEG), and in particular evoked responses, have long been used to investigate relationships between neural activity, human cognition, and clinical disorders^1,2^. Early investigations of stimulus driven activity reported transient ‘on-effects’ evoked by lights or sounds^3^. By the 1960s, this kind of work had evolved into what are now recognizable conventions and experiment designs for event-related potentials (ERP), including averaging over trials and the often used convention of labelling the neural responses by their polarity and latency (for example, ‘P100’ meaning a positive (P) potential peaking at approximately 100 ms). As well as further descriptions of early sensory responses^4^, ERPs started to be examined in relation to cognitive concepts, such as expectation, including early reports on the contingent-negative variation (CNV)^5^, the P300^6^, and the N400^7^. Other early work sought to investigate potential differences in ERP measures in clinical cases^8^.

**Figure 1:**
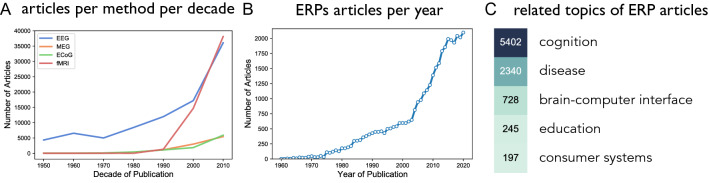
Prevalence of ERP research. (**A**) The number of articles across different methods for measuring brain activity, across decades. Note that each x-axis label reflects a decade (eg. “1950” reflects 1950–1959). (**B**) The number of articles on the topic of ERPs per year, measured as papers using the terms "event related potential", “ERP”, or “evoked potential”. (**C**) The number of ERP articles related to different topics, based on co-occurrence of ERP terms and listed association terms.

Since these early reports, investigating evoked electrical activity has continued to be a common and important method in cognitive and clinical studies. A large body of work has used ERPs to investigate the temporal dynamics of neural processing in perception and cognition^9–11^ and to investigate variations of brain responses in neuropsychiatric disorders^12–14^. The number of investigations using EEG, and specifically ERP designs, continues to increase (see Fig. 1A,B), with over 2000 ERP-related articles published in the year 2020. The extensive amount of work relating to ERPs also increasingly extends into applied areas such as brain-computer interfaces (BCIs) and consumer systems (see Fig. 1C).

The use of EEG and ERP designs remains appealing due to a combination of useful features, including that they: (i) offer direct measures of neural activity; (ii) have high temporal resolution; (iii) can be relatively easily measured across different populations and task designs and (iv) are low cost. With significant development in EEG hardware, leading to smaller and cheaper systems, the ease and use of EEG and ERP designs are likely to continue to grow. New and emerging applications of ERP analyses include deployment in real-world scenarios, for example in classroom settings^15^, and large-scale data collection and analysis, for example, data from thousands of subjects collected using consumer systems^16^. These new opportunities and applications are also engaging new groups of researchers, clinicians, and even home users, who all need tractable and understandable information about the current status of the field and up to date summaries of existing research.

The history and popularity of ERP research has created a catalog of literature that is prohibitively large for individuals to read and keep up to date with. The breadth of the literature can also be intimidating for newcomers to the field. In addition, important and potentially illuminating synthesis work connecting between ideas and experiments is difficult to pursue. Existing approaches for addressing large collections of literature include systematic meta-analyses, and review articles. However, these endeavors are labor-intensive and typically only include subsets of the literature, focused on specific issues and ideas. Therefore, aggregation work tends to lag behind the primary literature and is often incomplete.

The field of informatics, generally defined as approaches for storing, manipulating, and summarizing data and/or information, has been adopted by other areas of neuroscience and biology to address problems of scale and organization of concepts and data. Related work in informatics includes literature-based discovery and hypothesis generation, in which databases of literature are used to curate knowledge, annotate terms, and infer data-driven hypotheses, often based on relatively simple term co-occurrence measures^17–19^. Such approaches have been employed in the biomedical literature^20^ including within neuroscience, for example the NeuroSynth tool^21^ for functional MRI, and the 'BrainSCANR' project which analyzed patterns of associations in order to generate potential novel hypotheses^22^. However, there has been a relative lack of such work applied to EEG/ERP research, and there is no, to our knowledge, systematic, literature wide, attempt to analyze or curate the existing ERP literature.

**Figure 2:**
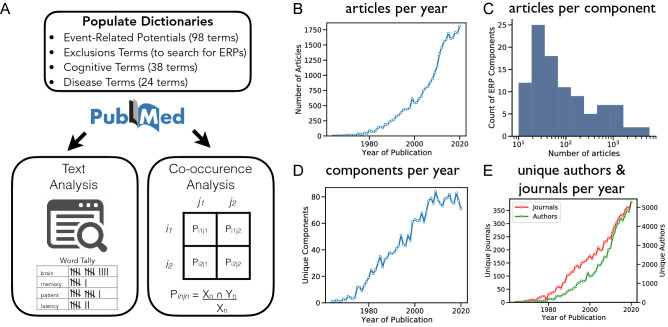
Data collection and dataset description. (**A**) Schematic of the data collection and analysis. **B**) The number of ERP articles per year, collapsed across all components. **C**) The distribution of the number of articles found across ERP components. (**D**) The number of unique components that are studied per year. (**E**) The number of unique journals and authors per year in the collected dataset.

Here we propose and demonstrate an automated meta-analytic tool for ERP-related literature. By collecting and analyzing relevant articles, we explore associations within and between ERP components and associated topics (see Fig. 2A). To do so, we systematically collected information from the PubMed database, selecting articles that discuss ERPs. We then build data-driven profiles for individual ERP components and analyze across component profiles to summarize the current state of the literature. The results from this analysis serve as an efficient data-driven summary of the extant literature, that can be automatically updated as the literature expands. We also explore patterns of similarity and differences across ERP components and associations, exploring how these patterns may be useful for knowledge discovery and highlighting patterns across the literature that are not evident from individual works. This data-driven procedure can also be useful to examine the current status of the nomenclature of ERP components, annotating putative associations, and motivating future work to critically examine and curate the ontology of ERP components and associations.

## Methods

For this study, data and metadata from scientific articles discussing ERP related research was collected and analyzed. To find relevant articles, dictionaries of terms reflecting known ERP components and potential association terms were curated to use as search terms. These search terms were used with two complimentary approaches for collecting and analyzing the existing literature. In the first approach, the ERP search terms were used to find relevant articles, from which text and metadata were collected. From this data, we can construct data-driven profiles for each ERP component to analyze general properties of the literature. In the second approach, based on word co-occurrence, separate searches were used to systematically count the number of articles in which curated lists of ERP terms, cognitive terms, and disorder-related terms co-occur. These co-occurrence measures can be used as an index of associations between ERPs and their putative cognitive and clinical correlates.

The *lisc* module, which provides programmatic access to available literature databases by integrating with available application programming interfaces (APIs), was used for collecting and analyzing literature data^23^. In addition, the scipy toolbox was used for some analyses^24^, and the matplotlib toolbox was used for creating figures^25^. All code for this project was written in the Python programming language (version 3.8) and is openly available in the project repository (https://github.com/ERPscanr/ERPscanr). The project is also hosted on a project website (https://erpscanr.github.io/). All data collection and analyses were carried out in accordance with relevant guidelines and regulations.

To estimate the prevalence of ERP related research, we ran literature searches collecting the number of articles across time. First, to compare ERP work to other modalities, literature searches collected the number of papers per decade for recording modalities [‘EEG’, ‘MEG’, ‘ECoG/iEEG’, ‘fMRI’]. To then estimate the prevalence of ERP research, we collected counts, per year (1960–2020), of papers using the terms [‘event related potential’, ‘ERP’, ‘evoked potential’]. Finally, to estimate which topics are investigated with ERPs, we collected the number of papers containing both the ERP search terms, as well as association terms ['cognition’, ‘biomarker’, ‘brain computer interface’, ‘education’, ‘consumer’]. Note that these collections, presented in Fig. 1, examine the field of ERP research as a whole, and are distinct from all subsequent literature searches which are specific to individual ERP components.

To examine individual ERP components, an exhaustive list of component names and synonyms was manually curated, including 98 ERP components. Where ERPs have names other than the P/N### convention, the full name of the ERP was used. Where names of ERPs have multiple variations, these different names were used together as synonyms (using the ‘OR’ operator). Early sensory components (onset prior to 100 ms post-stimulus) were excluded, to focus the analysis on cognitive components. Potential components were not included if they returned fewer than 10 articles in initial searches due to the limited utility of an automated summary of such a small number of papers, though there would not be any technical limitation to including them. We also curated lists of exclusion terms to exclude articles using synonyms of ERP component names reflecting unrelated topics. For example, since 'P100' can also refer to an antibody complex, an exclusion word of 'antibody' or similar can be used to exclude search results in which both the terms 'P100' and 'antibody' occur in order to exclude articles not related to ERPs. Due to the idiosyncratic nature of if and when ERP terms had synonyms, exclusion terms were individualized for each ERP component, based on examining search results.

For the co-occurrence analysis, lists of cognitive-related and disorder-related terms were also manually curated. Association terms were sub-selected from the Cognitive Atlas, a proposed ontology for cognitive neuroscience^26^. The ‘cognitive’ category of terms includes broad categories of perceptual and cognitive related terms (example: ‘vision’, ‘attention’, ‘conflict’). The ‘disorders’ category of terms includes both psychiatric and neurological disorders and impairments (example: ‘dementia’, ‘ADHD’, ‘schizophrenia’). Cognitive and disorder terms that were deemed synonymous were also used together with the 'OR' operator. In total, 38 cognitive and 24 disorder-related association terms were used. The full lists of search terms for ERPs, cognitive terms, and disorder terms, including exclusion terms and components that were dropped from the analysis, are available in the project repository.

All literature data was collected using the E-utilities API, which provides programmatic access to the PubMed database. PubMed is a database maintained by the National Centre for Biotechnology Information (NCBI), that provides searchable access to a bibliographic database of biomedical literature. The 'ESearch' utility was used to find IDs for relevant articles, as well as to extract the number of articles that contain specified terms, and combinations thereof. The 'EFetch' utility was used to retrieve data for identified articles. For each data collection that was initiated, the 'EInfo' utility was used to collect metadata about the version of the database that was accessed. All collections used the PubMed database (`db = pubmed`), and searched for terms of interest in the title and abstracts (`field = TIAB`).

An initial data collection procedure was used to collect information from all articles identified that contain any of the ERP terms. In the first step, we used an 'ESearch' call with each ERP term, including the exclusion words but with no cognitive or disorder terms, and collected all the article identification numbers for all articles responsive to the search query. We then ran an exhaustive set of 'EFetch' calls, to return each article identified by the search call. The PubMed database that we used does not include full-text articles, and so the extracted data is limited to abstract text and metadata. For efficiency, these searches used interim storage of intermediary results on the EUtilities server (‘usehistory = y’). Results were returned as structured XML files (‘retype = xml’), and subsequently parsed into fields of interest. For each identified article, we collected (if available), PubMed ID, DOI, title, authors, abstract text, year of publication, journal of publication, and associated keywords. In cases in which some information was not available in the record, missing values were stored as None.

Using the collected text and metadata, data-driven profiles were created for each ERP component. To do so, we used the collected text data, and computed summary measures across collected features, including the total number of articles, the number of publications per year, the number of publications per journal, the number of publications per author, the most common keywords, and the distributions of words used in the abstract text. For all text fields, pre-processing procedures included tokenizing the text (splitting up text into individual words) and removing stop-words (common words such as ‘a’, ‘the’, and ‘to’ that don’t add information about the content of the text). Each measure was calculated separately for each ERP term, and the results were used to create a data-driven profile per ERP component.

To investigate the overlap in articles discussing multiple ERP components, we did a network analysis using a non-directed weighted graph. For this analysis, the dataset was restricted to ERP components with at least 150 articles, leaving 31 components. To create the network, each node was an ERP component, and edge weights between nodes were calculated as the number of articles that mention both components, normalized by the total number of unique articles across both components. Standard network measures, such as node degree, defined as the number of connections each node has, eccentricity, defined as the maximum distance from one node to all other nodes, and average shortest path length, defined as the average number of steps along the shortest paths for all pairs of nodes, were computed to characterize the network. Network creation, analysis, and visualization was done using the `networkx` module^27^.

In a separate data collection procedure, the PubMed E-utilities were used to collect term co-occurrence between ERP components and cognitive and disorder terms, using the 'ESearch' utility. An example search, for ERP component 'P300', exclusion word 'protein' and cognitive term 'attention' would look like `"P300"NOT"protein"AND"attention"`. This search term returns information including the number of articles found with such a combination of terms, which was collected and stored. To collect normalization data, we also ran searches and extracted the number of articles for each ERP, cognitive, and disorder term in isolation.

The co-occurrence data collection created a dataset consisting of the number of co-occurrences between each ERP term and each association term of interest, for each set of association terms (cognitive and disorder-related). This data is encoded as the number of articles that contain both a given ERP component and another term of interest, which can be written as:

$$|ER{P}_{i}\cap ASSO{C}_{j}|$$

where | | is the magnitude (count) and $\cap$ is the intersection (co-occurrence) for each ERP term i and association term j. To obtain a relative measure, normalizing across the different number of articles addressing each term, we normalized counts across each ERP by dividing by the total number of articles found for that ERP term, as:

$$\left|ER{P}_{i}\cap ASSO{C}_{j}\right| / |ER{P}_{i}|$$

Through this procedure, we obtain a counts matrix, C, where each C_ij_ represents the proportion of all articles containing ERP term i that also contain term of interest j, for each set of associations.

Once the data is represented as above, we can consider the row of values for each ERP as a feature vector representing an ERP component in terms of relative proportions of articles about that component that also discuss each association term of interest. Since each ERP term is defined with a common feature space (the same set of association terms), this then allows us to systematically analyze patterns across ERPs. Specifically, we can compare the similarity of ERPs by calculating the pairwise distances between each feature vector, sort for similarity, and perform clustering. The distance between any two ERPs was calculated as the cosine distance between their vectors of literature defined word co-occurrences, as this distance measure works well with the high-dimensionality and scale variance of term-based feature vectors. Once this distance was calculated between each pair of ERPs, the feature matrix can be re-ordered to reflect the similarity between ERPs and terms of interest, upon which we performed hierarchical clustering, using the Farthest Point algorithm (see ‘scipy.cluster.hierarchy.linkage’ for details).

Finally, to investigate the temporal dynamics of neural processing, as assessed by ERPs, we analyzed the primary associations of each ERP, organized based on the canonical post-stimulus timing for each ERP component. To do so, we first labelled each ERP with its typical post-stimulus latency. We then associated each ERP component with its primary cognitive association, from the co-occurrence analysis. Doing so allowed us to examine the primary associations of ERPs across time. For the visualization only, this analysis was restricted to ERP components with at least 250 articles. We further sought to examine if ERP latency is related to the general strength of cognitive associations of the component. To do so, we calculated the median association strength, across all cognitive correlates, per component. To examine if there was an association between median association value and latency, we calculated the spearman correlation between the two. This analysis was done on ERP components having at least 50 papers (52 components), with results being qualitatively similar if different inclusion thresholds are used.

## Results

In the literature collection, 31,556 articles were identified across all 98 ERP components, reflecting publications from between 1964 and 2021. Note that individual articles could be included multiple times across different components. To examine the number of unique articles in the data, we compared digital object identifiers (DOIs), and found that this collection represents 21,579 unique articles. Across all components, we find that ERP experiments continue to be a highly prevalent method, with the number of ERP articles per year continuing to grow (Fig. 2B). The distribution of ERP related articles is significantly skewed (Fig. 2C), whereby a small number of ERP components are heavily studied while the majority of components are the subject of only a relatively small number of articles. The number of unique components present per year (Fig. 2D), as well as the number of unique authors and journals per year (Fig. 2E) are increasing, consistent with ERPs being a research topic that continues to grow.

Across the collected literature data, there is a large variety of topics. For articles with keywords (34.36% of articles), there were 14,203 unique keywords, including 753 that appeared at least 10 times. ERP articles have been published in a large number of journals, with 2106 unique journals, including 330 journals with at least 10 articles. The literature is also created by a large number of individuals, with 43,366 unique authors in the collection, including 2498 who co-authored at least 10 articles.

**Figure 3:**
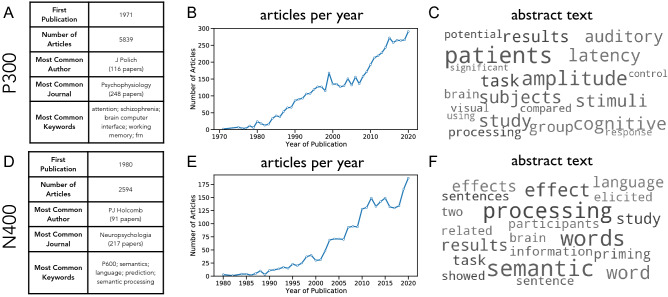
Example ERP profiles. Profiles for the P300 (**A**–**C**) and N400 (**D**–**F**) are created from text and metadata collected from all identified articles related to these components. (**A**) Summary statistics and metadata about articles on the topic of the P300. (**B**) The number of articles published per year for the P300. (**C**) The distribution of the most common words from the abstracts of articles about the P300, represented as a wordcloud. (**D**–**F**) Same as (**A**–**C**), but for the N400. Summaries for all ERP components are available on the project website (https://erpscanr.github.io/).

We created data-driven profiles for each ERP component, describing its associations and prevalence throughout the literature. These profiles show the publication history (number of articles across years), descriptive summary measures of the literature (including number of articles, common journals, authors and keywords), and a representation of the most common words used in articles discussing this ERP component, reflecting the main topics of investigation. Example profiles for the P300 and N400 are shown in Fig. 3, showing, for example, that both of these components are heavily studied, with increasing numbers of publications, and that the P300 is associated with visual and auditory stimuli, while the N400 is more associated with language and semantic related topics. The data-driven profiles for all collected components can be explored on the project website (https://erpscanr.github.io/).

**Figure 4:**
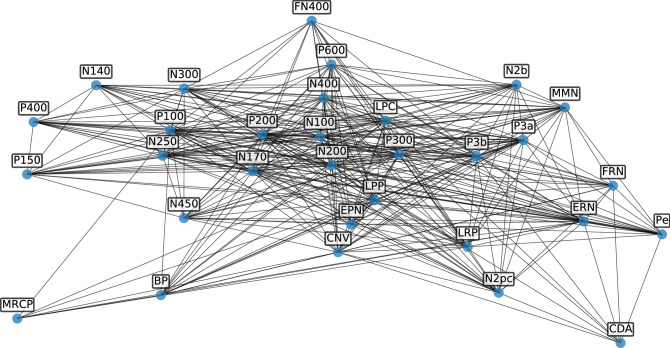
ERP network. A network representation of the ERP literature, in which each node is an ERP component (sub-selected for components with at least 150 articles) and each edge is a weighted connection based on the number of articles discussing both components together.

We also analyzed the network structure of the ERP literature, defined in terms of articles that discuss multiple ERP components (Fig. 4). In this network, each node is an ERP component, and each weighted edge is scaled by the number of publications discussing both components. Notable strong connections can be seen, for example, between the P600 and N400, and between the error related negativity (ERN) and the error related positivity (Pe). This analysis shows that the ERP literature is highly interconnected, which can also be seen in the average degree, which reflects the average number of connections of each node, of 22 (out of 30 components included in this analysis based on having at least 150 articles). This network has an average shortest path length between nodes of 1.27, and average eccentricity (maximum distance from the node to other nodes) of 1.97. This reflects a highly interconnected network, which is also seen in the network having 338 out of 465 possible edges, being 72.69% connected. The distribution of weights is highly skewed (approximately log-normal), indicating that most components are lightly associated (sometimes discussed together), with a small number of heavily associated components that are often discussed together. These measures are qualitatively consistent if the minimum number of articles required to be included in this analysis is changed or removed.

Subsequent analyses used the separate co-occurrence data collection. In this collection, across the 98 components, a total of 31,635 (potentially non-unique) ERP related articles were identified, with a skewed distribution across components, matching the data collected in prior analyses. In total, 8,609,245 articles across the 38 cognitive association terms were identified, with a total of 80,084 ERP-cognitive term co-occurrences. For the disorder related association terms, a total of 2,244,195 articles were identified across the 24 terms, for a total of 9256 ERP-disorder term co-occurrences. Overall, cognitive associations were more common, with 67.13% of possible co-occurrences in the cognitive data being non-zero, whereas only 34.99% of disorder co-occurrences were non-zero.

**Figure 5:**
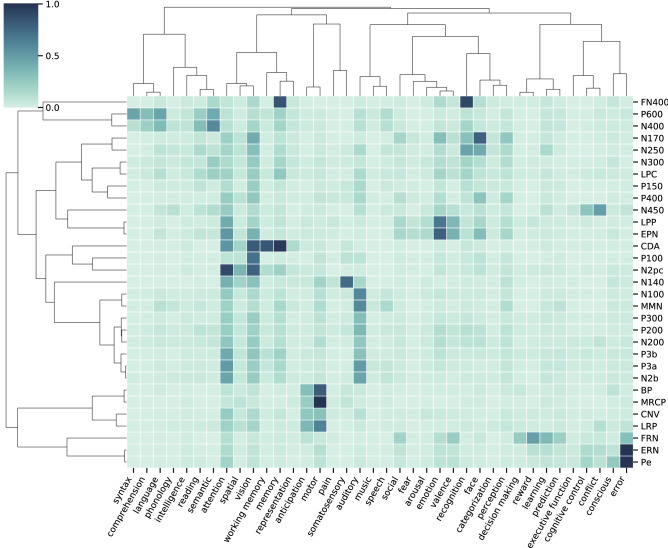
Cognitive-related associations. The heatmap is the normalized co-occurrence data, reflecting the percentage of articles for a given ERP that mention a particular cognitive term. Rows and columns have been re-ordered based on the similarity between ERPs and cognitive terms respectively. Dendrograms are hierarchical clustering based on cosine similarity. Note that only ERP components with at least 150 articles are included in this analysis. Figure created with the lisc Python package, version 0.2.0 (https://lisc-tools.github.io/).

**Figure 6:**
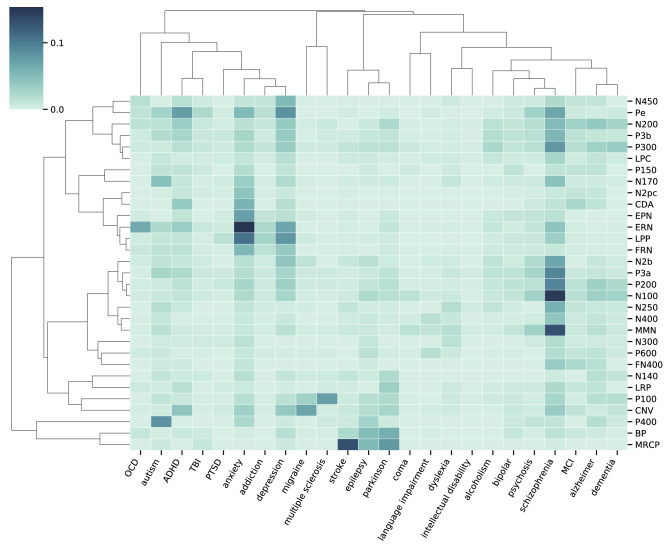
Disorder-related associations. The heatmap is the normalized co-occurrence data, reflecting the percentage of articles for a given ERP component that mention a particular disorder-related association term. Rows and columns have been re-ordered based on the similarity between ERPs and disorder terms respectively. Dendrograms are hierarchical clustering based on cosine similarity. Note that only ERP components with at least 150 articles are included in this analysis. Figure created with the lisc Python package, version 0.2.0 (https://lisc-tools.github.io/).

To examine the structure of associations in the literature, we visualized the co-occurrence data across all ERP components together, computed similarity measures using cosine similarity, and re-ordered components based on hierarchical clustering. For these analyses, the dataset was restricted to components with at least 150 articles per ERP component. This approach highlights the structure across the literature and provides a quantitative estimate of the similarity between all ERP components. Notably, in both the cognitive related (Fig. 5) and the disorder-related co-occurrences (Fig. 6), the clustering of the association terms (based on their associations with ERP components) organizes terms together into related sub-groupings (for example ‘fear’ clustering with ‘arousal’ and ‘Alzheimer’s’ clustering with ‘dementia’), consistent with this analysis being able to find meaningful structure in the data.

In the cognitive-related association data (Fig. 5), ERP components show clustering of similar components, such as the P3a and P3b, and Bereitschaftpotential (BP) and movement related cortical potential (MRCP) clustering together. Although clustering of components sometimes follows latency, for example the N200, P200, and P300 clustering together, latency does not seem to explain all the similarities. Rather, this analysis groups components into groupings most related to particular cognitive concepts. For example, components such as the N400 and P600 cluster together, which have high associations with cognitive terms ‘semantics’ and ‘syntax’, and the N170 and N250 cluster together with a strong association to face processing. Notably, some components whose time course is similar, such as the N400, FN400, and N450 or the N2b and N2pc are found to have quite distinct patterns of association, suggesting they are discussed quite differently in the literature.

In the disorder-related association data (Fig. 6), ERP components are also clustered together, in a broadly consistent manner as in the cognitive-related association data. Clear clusters of association include that anxiety has high association with error related components such as the error related negativity (ERN), stroke and Parkinson’s is associated with movement related components such as the Bereitschaftspotential, and schizophrenia has relatively high associations with a number of components, including components related to stimulus predictability and sensory processing, such as the N100, and mismatch negativity (MMN). As with the cognitive associations, though some temporally adjacent components cluster together, for example the P200 and P300, temporal adjacency doesn’t appear to explain the clustering overall, with some temporally distinct ERP components being grouped together based on their relation to disorders, such as the N300 clustering quite closely with the P600.

**Figure 7:**
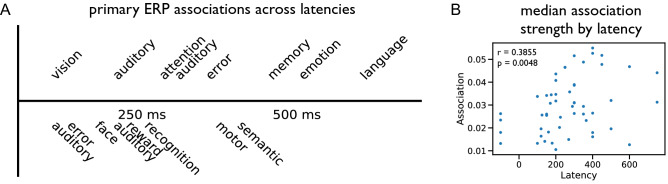
ERP associations across latencies. (**A**) Primary associations for each ERP were extracted from the co-occurrence analysis, and ERPs were plotted across time based on their typical peak latency. In general, earlier components tend to reflect sensory processing, whereas later components increasingly relate to more cognitive processes. For visualization purposes, only components with more than 250 articles are shown in this figure. (**B**) The median association value per ERP component is positively correlated with the typical peak latency of the ERP component, suggesting that ERPs with later latencies are more broadly associated to cognitive correlates. The inset text reports the r-value and associated p-value of the Spearman correlation measured between the latency and association values.

Finally, we examined the primary association of ERP components across time, by ordering components by their canonical post-event time of occurrence (latency) and extracting the most highly associated cognitive term (Fig. 7A). This analysis found that earlier components tend to reflect sensory processes (e.g., vision, auditory), whereas later components increasingly related to more cognitive aspects (e.g., emotion, language). We also found a positive correlation between ERP components average cognitive association score and peak latency (Fig. 7B), suggesting that later ERP components are on average discussed with a broader set of cognitive correlates.

## Discussion

With a large existing literature and an expanding contemporary user base, there is a need for systematic and scalable tools for dealing with the increasing volume of research on the topic of ERPs. Here, we partially address this need by developing and applying an automated approach for systematically analyzing the extant literature. In doing so, this project offers a data-driven view of the status of the field, summarizing current knowledge and patterns in the literature. This information can serve as a pedagogical tool for researchers, and particularly newcomers to the field, offering summary reports of each examined ERP component (Fig. 3) as well as overviews of how ERP components are associated with cognitive processes (Fig. 5) and disorders (Fig. 6). To facilitate this kind of usage, and allow for full exploration of the data, the collected data is made fully available, and all results are openly available on a public website (https://erpscanr.github.io/).

This kind of automated meta-analysis also serves as an opportunity to highlight themes in the literature in order to inform future work that seeks to investigate relationships between evoked brain activity and cognitive and disorder-related associations. This tool may be particularly useful for newcomers to the field to explore a summary of the literature, and to direct them to relevant components given a topic of interest. For expert researchers, though many of the themes highlighted by this analysis may be familiar, with almost one hundred ERP components examined across more than 20,000 unique articles, these results may be useful to examine topics outside of one’s core expertise and highlight previously missed associations. This kind of investigation also allows for meta-analysis beyond what is possible within individual studies, investigating, for example, the time course of cognitive processing across all ERP components (Fig. 7) showing how temporal adjacency potentially explains some similarity across components. With an ever-growing literature, another benefit of the current approach is that, due to using an automated pipeline, analyses can easily be periodically updated to integrate new information from the literature.

In collecting the search terms, we identified many ERP components that are currently discussed in the literature. When interpreting these findings, it is important to consider that distinct labels do not necessarily imply that each component is a distinct physiological event. However, it is difficult to know if and when these components are unique, and/or are synonymous labels for the same underlying process. Diverse fields of inquiry use ERP methodology—ranging from clinical practice to linguistics, cognitive psychology, and brain-computer interfaces—and they do so using idiosyncratic language to describe both task designs and physiological findings. This variability in naming means that consistencies across domains may be obfuscated by terminology differences. Relatedly, this analysis cannot definitively adjudicate whether labels refer to the same or distinct processes, as the (dis)-similarity of literature data, as used here, can only indicate the similarity of the research about a component, and the way it is described, and does not directly examine the properties of the physiological events themselves.

This project raises overarching questions about the field of ERP research, including about the ‘ontology’ of ERPs—asking how many distinct ERP components there are, how they relate to each other, and what cognitive processes and disease states they relate to. Ontologies have been highly valuable in other scientific fields, perhaps most notably in genetics, in which the gene ontology provides a common naming structure and system for defining entities of interest (genes) and their properties within genetic research^28^. Ontologies have also been proposed within neuroscience, for example the NeuroLex ontology for neurobiological knowledge^29^. In the domain of ERPs, the neural electromagnetic ontologies (NEMO) have previously been proposed as a set of tools and approaches to develop an ERP ontology^30,31^. Notably, their approach to defining a specific analysis procedure for empirical data is quite different to the literature-based approach taken here, and though the project has overlapping goals, the NEMO approach does not appear to have been integrated into research practice.

The need for a consistent ontology of ERP components includes scientific goals for systematically organizing the knowledge collected across thousands of investigations, as well as practical elements that can assist with navigating the literature. For example, the list of ERP components curated here reflects multiple naming schemes, including by polarity and specific latency (e.g., ‘N400’), by broader physiological description (e.g., ‘late positive component’), or by cognitive association (e.g., ‘error related negativity’). Such a lack of systematicity in ERP naming and reporting makes it difficult to both compare between studies using different schemes and to integrate knowledge into a broader understanding of neural processing. Ultimately, we would like a more unified understanding of ERPs, including understanding if individual components reflect independent processes and/or to what extent there are related physiological processes engaged in similar ways across different tasks and contexts that may reflect, in at least some cases, continuous variation. This project demonstrates the utility of text-mining approaches beyond simply describing what has already been done, showing that the huge amounts of data can be leveraged to uncover “hidden” information across the entirety of the literature^32^.

By curating information about ERP-related research, the current project provides a potential starting point for future work designed to systematically curate an 'ontology' for ERP research, including standardizing a naming scheme, identifying synonyms, and mapping associations. Using collections of systematically defined physiological terms, as done, for example, in clinical EEG^33^, and integrating these with ontologies of cognitive process and their relation to tasks^26^ provides an opportunity for moving towards a more formal ontology of ERPs. Employing these strategies must also include using agreed upon standards and nomenclature for discussing ERP related findings^34^, as well as community standards for transparent and reproducible analysis approaches^35^.

ERP research is an example area in which automated approaches for curating and summarizing the literature may help address the growing scale of the literature. Investigations of ERPs are relatively amenable to such an analysis since the names of ERPs components offer relatively consistent keywords for literature searches. This consistency makes the field a productive case study for applying automated meta-analytic approaches, informatics, and formal ontologies that may also be usefully applied to other areas in cognitive neuroscience^36^. The field of bioinformatics offers tools and relevant examples^20^, for example, fields such as genetics, in which there is a relatively clear set of elements that one wants to curate information about, and a productive system for developing field level ontologies^28^. Future work should explore how other areas of cognitive neuroscience could potentially benefit from similar informatics work. In doing so, potential issues such as ambiguous term use and inconsistent ontologies of concepts that makes automated tagging difficult will need to be addressed^34^. Addressing these issues will require investing in developing and systematizing consistent ontologies and term usage within and across fields^26,37^, as well as using machine readable formats^38^, such that automated measures can be used to address literatures that continue to grow in both size and complexity.

There are limitations to literature analyses, including being constrained by the curated search terms used to collect the data. Though we attempted to exhaustively list known components, only components with conventional naming schemes are included, which may limit results from, for example, older articles that predate these naming conventions, and/or reports employing novel and idiosyncratic component names. Different component names require different amounts of manually tuned exclusion terms, and variance in the efficacy of exclusion words for excluding irrelevant articles may add some noise to the data. Additionally, all articles including specified search terms are treated as an equivalent single data point, with no separation of primary research reports from review articles, and no weighting or exclusion of reports based on the number of experiments, sample sizes, or quality control metrics. Articles are also only included if search terms occur in the abstract or title, which highly impacts the data selection procedure, suggesting that while included articles are likely highly relevant to search terms, many other relevant articles may be missed.

A key consideration concerning literature analyses is that by using articles as data, the analysis is constrained to examining how entities of interest are discussed in the literature (rather than investigating properties of the entities of interest directly) and is thus liable to biases in research topics and publication bias. The analyses of literature data employed here are coarse measures, applied at scale to identify general patterns, and do not include the rich detail available in individual articles. For example, analyses such as term co-occurrence merely imply that topics are discussed together, and does not specify how, or even if, these topics are actually related. Overall, while this approach is useful for capturing general patterns at scale in the literature, it does not allow for nuanced appraisal of how things are discussed. Future work that continues to develop ERP ontologies and literature annotation to assist with automated searches, as well as integrating more detailed analyses and including full text data, may help address these limitations.

Despite these limitations, large-scale text analyses have been productively applied to characterize fields of inquiry^20,22,39,40^, specific topics of interest^41,42^ and meta-science questions such as citation patterns^43^ and science communication^44^. Each of these investigations help to summarize current knowledge and to highlight novel findings from analyses at scale that are otherwise unacknowledged in the literature. As journals and publishers continue to make full scientific texts more open and freely available, the capacity for using aggregate scientific articles as data, and extracting more information, is also increasing^45,46^. As the literature continues to expand, using literature-based analyses and informatics approaches offers strategies for cognitive neuroscience to continue to organize existing knowledge, discover new findings, and work towards systematic and scalable tools for investigating brain and behavior.

## Conclusion

ERPs are a common method for investigating neural activity and its relation to cognition and disease. In an attempt to explore and summarize the large existing literature, this study consists of a proof-of-concept automated meta-analysis of the ERP literature. With a publicly available dataset and hosted website, this project allows for systematically exploring the current state of the ERP literature, highlighting patterns, and summarizing what is known about many ERP components, as well as allowing for novel analyses across the entire literature. This project may serve as a pedagogical tool for researchers to explore associations in the literature in order to develop and pursue novel hypotheses. This project also serves as a demonstration and motivating factor for developing and applying systematic ontologies and practical informatics approaches in cognitive neuroscience.

## Data Availability

This project uses literature data collected from the PubMed database. The data collected from the literature and metadata about the collection are saved and available in the data repository (https://osf.io/g2ruj/). In addition, the code and search terms used to collect the data, which can be used to re-run the data collection, are available in the project repository (https://github.com/ERPscanr/ERPscanr). The full set of processed results, including the computed profiles for each component are also available on a project website (https://erpscanr.github.io/).
